# Where Student Teachers Struggle: Scoring Accuracy and Feedback Quality as a Function of Text Level, Rubric Criteria, and Teacher Traits

**DOI:** 10.3390/bs16101927

**Published:** 2026-10-17

**Authors:** Hyeyoun Kim

**Affiliations:** Department of Korean Language Education, Dongguk University, Seoul 04620, Republic of Korea; hyeyoun.kim@dgu.ac.kr

**Keywords:** writing assessment, pre-service teachers, rater cognition, evaluative judgment, scoring accuracy, feedback quality, individual differences, cognitive empathy, cognitive load

## Abstract

Although student teachers often report low confidence in writing assessments, empirical evidence regarding the behavioral and cognitive mechanisms affecting their evaluative judgments remains limited. This study investigated the evaluative behavior of 161 student teachers, yielding 1932 scoring observations and 2415 coded feedback segments. Fluctuations in scoring accuracy and feedback quality were examined as a function of text proficiency levels, scoring criteria, and feedback dimensions. Participants evaluated three Grade 9 argumentative texts (low-, mid-, and high-level) using a four-criterion analytic rubric. Participants’ writing quality and empathy (cognitive and affective) were analyzed as potential moderators. Linear mixed-effects models revealed that scoring accuracy is a function of the interaction between text proficiency level and criterion: accuracy was highest for focus in high-level texts and organization in low-level texts. Feedback quality also varied significantly across dimensions: evaluation quality and rubric-aligned explanations were weakest for mid-range texts, reflecting potential evaluative strain when navigating text ambiguity. While student-teachers’ writing quality showed no significant effects, their cognitive empathy moderated text-level effects and positively predicted feedback quality. These findings illuminate the psychological underpinnings of rater cognition, providing a foundation for targeted pedagogical interventions in teacher education programs.

## 1. Introduction

Writing assessment is a high-stakes, cognitively demanding task that requires assessors to simultaneously process multiple textual dimensions (e.g., content, organization, and language use) while diagnosing student writing needs (Weigle, 2002). While experienced teachers utilize highly automated pedagogical schemas to optimize this complex process, student teachers, given their developing assessment literacy, generally experience severe difficulties in writing assessments (Crusan et al., 2016; Hodges et al., 2019). Student teachers often report low confidence in assessment (Hodges et al., 2021); however, empirical research directly examining the concrete difficulties and challenges they face when performing actual scoring and feedback tasks remains notably scarce.

### 1.1. Scoring Accuracy and Cognitive Ambiguity

Writing scores are prone to vary by rater (East, 2009; Lumley & McNamara, 1995). This rater influence is particularly pronounced in analytic scoring (Alaei et al., 2014). Consequently, two pivotal issues arise: how to reconcile discrepant scores through consensus procedures, and how consistently evaluations are made within and between raters (East, 2009). Synthesizing prior discussions, resolution models for addressing interrater score discrepancies can be categorized into five types: rater mean (averaging scores), parity (using a third rater to combine scores), tertium quid (selecting the score closest to a third rater’s assessment), expert judgment (replacing discrepant scores with an expert’s), and discussion (deliberation between raters to reach a consensus) (Johnson et al., 2005; Penny & Johnson, 2011; Sevgi-Sole & Ünaldı, 2020). Although many studies emphasize the importance of rater consensus, achieving agreement through equal participation of two raters does not necessarily yield a true score (Sevgi-Sole & Ünaldı, 2020).

To reduce threats to scoring accuracy, it is important to examine whether rating scales and their categories function as intended, because evidence about rating-scale functioning supports more valid, reliable, and fair interpretations of scores (Wang & Engelhard, 2026). From the perspective of scoring accuracy, the reference score for a piece of writing is typically determined in one of three ways (Sulsky & Balzer, 1988; Wang et al., 2017): (a) the grand mean of all participating raters’ scores, (b) pre-established benchmark scores assigned to standardized anchor texts, or (c) the target scores assigned by a panel of expert raters. Among them, scoring accuracy has most often been discussed in terms of discrepancies from expert-group scores (Engelhard, 1996; Wang et al., 2016, 2017). Since deviation from expert judgments serves as a suitable reference point for evaluating individuals who have not yet developed professional rater expertise, this criterion is particularly relevant for judging student teachers’ assessment literacy (Wang et al., 2016, 2017). Nevertheless, research remains limited not only on student teachers’ scoring accuracy but also on the evaluative practices that accompany their judgments.

To understand why raters differ even when they use the same rubric, researchers have increasingly examined the cognitive processes underlying scoring judgments. *Rater cognition* has been understood as a central component of writing assessment because raters do not merely apply scales mechanically; rather, they actively reconcile their overall impression of a text, its specific features, and the wording of the rubric (Lumley, 2002). Scoring is not a simple mechanical application of rubrics but involves complex psychological and cognitive information processing; thus, investigating rater cognition is essential to elucidate the internal mechanisms of evaluative judgment and enhance assessment validity and reliability (Gauthier et al., 2016; Huang, 2025). Moreover, the validity of resulting scores depends in part on whether this interpretive process remains aligned with the intended construct (Bejar, 2012). In writing assessment, this concern is especially salient because scoring criteria may be difficult to implement consistently, potentially collapsing multiple discourse, lexical, and rhetorical features into overly compressed scale points (Cumming et al., 2002; Tavazoei & Ahmadi, 2025), while raters’ individual reading styles and levels of scoring expertise may further shape how rubric information is encoded and applied (Wolfe et al., 1998). From this perspective, rating performance may vary not only because of text quality itself but also because raters rely on partially different mental scoring rubrics and judgment heuristics, particularly when texts present ambiguous or mixed signals that make criterion application less straightforward (Bejar, 2012).

This interpretive view of rating is also consistent with broader theoretical perspectives suggesting that human judgment often relies on internalized prototypes and becomes more difficult near category boundaries (Rosch, 1975; Andrich, 1978). In writing assessment, such difficulty may be especially pronounced when texts contain mixed or borderline features that do not clearly correspond to a single scale level. Under these conditions, raters may differ in how they prioritize salient textual cues and reconcile them with rubric descriptors, thereby increasing the likelihood of inconsistent or inaccurate judgments (Lumley, 2002). This suggests that even highly reliable raters are not merely scoring machines but active interpreters who may recontextualize rubric language to align with their internal perceptions (Chan, 2025).

Recent studies on rater cognition have increasingly moved beyond psychometric concerns with rater reliability, severity, and score consistency to examine the cognitive processes through which raters interpret task performances, attend to rubric dimensions, construct mental representations of texts or performances, and translate these judgments into scores (Han et al., 2025, 2024; Jadesi et al., 2022; Jia & Zhang, 2023; Tavassoli et al., 2022). This line of research has shown that rating is not a mechanical application of scoring criteria but a complex problem-solving process shaped by task type, rating scale structure, rater experience, and the interaction between raters and assessment materials.

Other researchers have investigated how differences in raters’ experience affect scoring decisions and related assessment practices (Barkaoui, 2010, 2011; Eckes & Jin, 2024; Tavassoli et al., 2022; Tavazoei & Ahmadi, 2025; Thai & Sheehan, 2022). Experienced and novice raters employ qualitatively different cognitive strategies: while experienced raters tend to be more cautious—collecting more information before arriving at a judgment and considering a wide variety of linguistic features—novice raters often exhibit category-specific bias (Barkaoui, 2010, 2011). The novices frequently focus disproportionately on lower-level features, such as grammatical accuracy or mechanics, at the expense of higher-order rhetorical concerns (Tavazoei & Ahmadi, 2025; Thai & Sheehan, 2022). Furthermore, novice raters are more susceptible to cognitive biases, such as the *halo effect*, where the judgment of one criterion (such as handwriting or grammar) unfairly influences others (Eckes & Jin, 2024; Tavassoli et al., 2022). These distinctions between experienced and novice raters provide critical insights for investigating the scoring patterns of student teachers.

### 1.2. Difficulties in Feedback Production

Teachers’ feedback on student writing has long been acknowledged as crucial—for offering individualized attention, integrating instruction, learning, and assessment, as well as for fostering genuine improvement in students’ abilities (Hyland & Hyland, 2006; Parr & Limbrick, 2010; Parr & Timperley, 2010). Since effective feedback should not simply correct errors but function as a form of instruction, many scholars conceptualize writing feedback as a pivotal stage that combines characteristics of teaching and assessment (Hattie & Timperley, 2007). Substantial research shows that the effects of feedback differ significantly by type: depending on its delivery, feedback can assist, impede, or even confuse writers (Bitchener et al., 2005; Brooks et al., 2021; Duijnhouwer et al., 2012; D. R. Ferris, 1997; Tunstall & Gsipps, 1996).

Many studies have expanded the possibilities of writing feedback by explicitly distinguishing between product-oriented and process-oriented approaches (Duijnhouwer et al., 2012; Hattie & Timperley, 2007; Hyland & Hyland, 2006; Liu & Yu, 2022; Vandermeulen et al., 2020). Product-oriented feedback traditionally adopts a summative stance, evaluating writing as a completed text with a primary focus on immediate text revision, linguistic accuracy, and surface-level quality. Conversely, process-oriented feedback takes a formative stance designed to guide students’ ongoing writing development, future drafts, and broader strategic growth (Duijnhouwer et al., 2012; Hyland & Hyland, 2006). Rather than positioning one approach as superior to the other, recent multi-dimensional frameworks emphasize that feedback operates across complementary domains—shaping immediate text quality (writing), procedural revision strategies (writing process), and long-term learner growth (writer) (Liu & Yu, 2022). From a similar perspective, Li and Collins’ study (Li & Collins, 2026) has marked a conceptual shift from the *feedback of learning* to *feedback for learning*, and further to *feedback as learning*, wherein learners proactively interpret feedback and self-regulate their own learning. Within this evolving paradigm, producing effective feedback extends far beyond simple scoring; it constitutes a distinct form of pedagogical decision-making—one that requires teachers to interpret student performance, prioritize instructional information, and translate evaluation into actionable guidance.

As such, providing feedback is a highly demanding cognitive task that requires simultaneous processing of student performance data, curriculum standards, and pedagogical strategies within a limited working memory. From the cognitive load theory (CLT) perspective, human working memory is severely limited, typically capable of holding and processing only a small number of information elements simultaneously (Sweller, 1988, 1994). Feedback production may therefore involve both intrinsic complexities, arising from the multi-dimensional nature of student performance, and extraneous demands, arising from contextual, interpersonal, and instructional pressures (Hyland & Hyland, 2006). This complexity is likely to be especially pronounced for student teachers, whose evaluative schemas and feedback routines are less consolidated and therefore may require greater cognitive effort to coordinate (Crusan et al., 2016; Ye et al., 2024). Examining teachers’ experiences through the lens of CLT, Alruwaili and Alasmari (2025) found that increased cognitive load constrained teachers’ capacity for responsive and engaging instruction, often leading them to simplify pedagogy and adjust their feedback-related interactions with students. This study illustrates that teachers’ feedback practices are partly shaped by how they manage their own cognitive burden within demanding instructional environments.

To support this demanding task, some research has aimed to enhance the quality of teacher response by developing ways to evaluate feedback quality and by proposing interventions to strengthen student teachers’ feedback skills. For example, building on Hattie and Timperley’s (2007) feed-up/feed-back/feed-forward framework, Parr and Timperley (2010) clarified the several kinds of information that effective responses should include as quality indicators: (a) where students currently are in relation to desired performance, (b) the defining characteristics of desired performance, and (c) what is required to achieve it. Grounded in these three theoretical directions, they operationalized feedback quality across five distinct dimensions—evaluation quality, accuracy of feedback, rubric alignment, textual scope, and actionable suggestions—to systematically evaluate raters’ formative responses. Further studies have established programs to advance student teachers’ feedback abilities (e.g., D. Ferris, 2007; Ye et al., 2024), including seminar-based training, feedback generation approaches that incorporate self-regulated learning, and school-based coaching in which in-service teachers mentor feedback practices directly.

Despite this progress, the field still lacks thorough investigation of student teachers’ actual assessment practices, training processes, and the barriers they encounter when producing feedback (Copland, 2010; Peck & Kavanagh, 2024). More fine-grained evidence is needed on student teachers’ actual feedback production processes, the cognitive and instructional barriers they encounter, and the conditions under which their feedback performance improves.

### 1.3. Contextual and Individual Factors Shaping Assessment

Although research on teachers’ scoring and feedback practices is relatively comprehensive, studies focusing on the factors that affect those practices are comparatively limited. In practice, teachers’ judgments about writing are shaped by a range of influences: individual and contextual characteristics, as well as features of the student texts themselves. For example, certain texts are more challenging to evaluate than others depending on their specific characteristics (Wolfe et al., 2016). It is often assumed that poorly written student texts are more likely to leave teachers at a loss, but there is little empirical evidence on how the difficulty of evaluation actually varies as a function of text quality level. Additionally, rater variability can change by scoring criterion—being smaller for some and larger for others (Humphry & Heldsinger, 2019). Nevertheless, little is known about whether student teachers’ particular challenges differ by text proficiency level or scoring criterion. At the same time, researchers have extensively investigated how student teachers’ prior knowledge and pedagogical content knowledge (PCK) influence their assessment practices (Carter et al., 2022; Glogger-Frey et al., 2018). Research on teacher cognition and literacy instruction demonstrates that teachers’ subject matter knowledge and personal practical theories directly shape how they observe student performance, interpret text features, and execute adaptive instructional and evaluative decisions (Lipka et al., 2025; Männikkö & Husu, 2019). However, to date, little attention has been devoted to the relationship between student teachers’ own writing proficiency and their competence in assessing writing. Thus, research gaps regarding writing assessment should be examined to determine whether personal domain execution directly translates into assessment competence or whether they function as empirically decoupled constructs.

Beyond contextual factors (e.g., assessment setting and task characteristics) and knowledge- and skill-based factors (e.g., teachers’ PCK and proficiency), teachers’ affective dispositions also deserve attention as potential influences on writing assessment. A substantial body of literature shows that teacher feedback can have emotional consequences for students (Brooks et al., 2021; Chang, 2015; Duijnhouwer et al., 2012; Hattie & Timperley, 2007; Hwang, 2025; Hyland & Hyland, 2006; Straub, 1996); however, relatively few studies have explored how teachers’ own emotional traits influence the feedback they produce (Yu et al., 2021). Although prior research has frequently examined how situational constraints such as time pressure, cognitive fatigue, and scoring contexts influence decision-making and evaluation (Guo & Wang, 2025; Palermo, 2022), empirical investigations into how raters’ individual affective traits directly shape scoring performance remain relatively scarce. However, teachers’ affective dispositions shape their work attitudes and routine instructional practices, with direct implications for student learning (Yu et al., 2021). Personality variables have been found to exert minimal influence on holistic scoring but notable effects in analytic scoring, where rater factors are more evident (Alaei et al., 2014). Consequently, teachers’ personality and affect—as well as their outward expressions of these states—may variably influence the effectiveness of both scoring and feedback.

Within the affective domain, several researchers have focused on teachers’ empathy and its links to performance. Empathy refers to an individual’s response to another person’s observed experiences and is commonly distinguished into two components: cognitive empathy, which involves adopting another person’s perspective, and affective empathy, which involves emotional responses to that person’s experiences (Davis, 1983; Reniers et al., 2011). However, prior research in other evaluative contexts offers mixed evidence regarding the impact of empathy. Specifically, while high empathy may compromise scoring objectivity in performance assessments (McNamara, 2000), low empathy tends to degrade feedback quality (Waung & Highhouse, 1997).

Within the educational context, various claims have also emerged regarding the extent to which teachers’ empathy influences their effective instruction and assessment. Alp et al. (2017) found that for raters with regular training and at least three years of experience, empathy levels did not significantly affect final scores. This aligns with the suggestion that rater effects, such as severity or leniency, are more likely products of inadequate training or a lack of self-consistency rather than a rater’s inherent empathic disposition (Fahim & Bijani, 2011). In contrast, Tettegah and Anderson (2007) emphasized the importance of empathy for student teachers and called for explicit empathy training. Assuming that effective feedback requires empathy, Lopez-Leon (2023) conceptualized it as a critical teacher competency and proposed a five-stage developmental model. Despite these theoretical advancements, research directly connecting teachers’ empathy with writing instruction or assessment remains scarce, underscoring the need for further investigation.

### 1.4. The Present Study

Based on the foregoing review, three critical research gaps and corresponding design considerations drive the present study. First, while writing assessment is recognized as a cognitively demanding task, it is essential to examine how student teachers’ scoring accuracy systematically fluctuates across rubric criteria and student text characteristics (e.g., text proficiency levels), capturing rater cognition under specific evaluative standards. Second, given the distinct pedagogical nature of feedback, a multi-dimensional evaluation of written feedback quality across text proficiency levels and feedback dimensions is necessary to understand how novice evaluators translate judgments into actionable guidance. Third, rater-level characteristics (e.g., writing proficiency and empathic dispositions) must be integrated into a unified framework to examine how individual traits moderate evaluative performance.

Accordingly, the present study investigates how the quality of student teachers’ scoring and feedback varies as a function of student text proficiency level and evaluative dimensions, while exploring the moderating roles of rater-level traits. The research questions are as follows:

- RQ1. To what extent does student teachers’ scoring accuracy systematically differ as a function of student text proficiency levels (low, middle, and high) and rubric criteria?
- RQ2. To what extent does the quality of student teachers’ written feedback systematically differ as a function of student text proficiency levels (low, middle, and high) and feedback dimensions?
- RQ3. How do student teachers’ individual traits (writing quality, cognitive empathy, and affective empathy) moderate the impact of text proficiency level ambiguity on scoring accuracy and feedback quality?

## 2. Materials and Methods

### 2.1. Participants

A total of 161 student teachers (109 women and 52 men; age *M* = 21.88 years, *SD* = 1.27) participated in this study. The participants were recruited from the department of education at a large comprehensive university in Seoul, South Korea. They were either third- (*n* = 117) or fourth-year (*n* = 44) students, recruited from language education majors (including double majors) who had completed coursework on the foundational theories of writing instruction. Regarding their assessment background, none of the 161 student teachers had completed formal, dedicated coursework in educational evaluation or writing assessment prior to participation. Their assessment knowledge was restricted to introductory concepts (e.g., general definitions, assessment types, and basic principles) covered within a general course, with no prior practical experience in evaluating student writing or providing formative feedback. Thus, participants represented novice assessors operating with limited, non-automated assessment schemas.

All participants were native Korean speakers, and the argumentative texts they assessed were written in Korean by native-speaking Grade 9 students (i.e., a first-language assessment context where both raters and student writers share the same native language). Five additional recruits were initially enrolled but excluded from the final sample due to ineligibility or discontinuation of participation. Using G*Power 3.1.9.7, the minimum sample size needed under a repeated-measures, within-factors design with α = 0.05, power = 0.80, and a small to medium effect size (*f* = 0.14) was calculated; results indicated that at least 57 participants (minimum 684 repeated observations) for scoring accuracy and 51 participants (minimum 765 repeated observations) for feedback quality were required. The study protocol received approval from the Institutional Review Board (IRB) of the author’s institution, and all participants provided written informed consent before data collection.

### 2.2. Research Design and Procedure

Before undertaking the main rating tasks, participants provided background data and completed two preparatory tasks to examine their personal characteristics: (a) an empathy questionnaire and (b) an argumentative writing task. These preparatory instruments were administered to measure key rater-level individual traits—specifically writing quality, cognitive empathy, and affective empathy—in order to examine how student teachers’ individual traits moderate the impact of text proficiency level ambiguity on scoring accuracy and feedback quality (RQ3).

Prior to the assessment task, participants viewed a standardized 97 min instructional video designed to calibrate baseline task understanding. The video content was organized into five sequential modules: concepts and objectives of writing assessment (foundational principles), holistic vs. analytic scoring methods (theoretical and practical distinctions), case-based rubric application (detailed explanation of the 4 analytic criteria with concrete scoring exemplars), principles and current status of written writing feedback (instructional significance of diagnostic comments), and case-based feedback modeling (demonstrating how to draft actionable, rubric-aligned feedback comments). This session covered the core concepts and precautions of writing assessment, analytic scoring, and feedback provision. Participants then evaluated and commented on samples of ninth-grade students’ argumentative writing with a standardized task prompt (Appendix A). They were given printed copies of the student texts and the analytic rubric and completed the scoring and feedback tasks during a 90 min session.

To manage participant fatigue and cognitive load, each student teacher evaluated a set of three representative argumentative essays—one from each proficiency level (low, middle, and high; examples in Appendix B)—selected from the initial pool of 78 ninth-grade essays based on expert consensus median scoring. The target texts were selected as follows. Initially, a total of 78 ninth-grade students recruited from a middle school in a metropolitan area wrote argumentative essays. They completed a 45 min pen-and-paper writing task in which they were asked to compose an essay regarding issues in school life or educational situations. After collecting these base materials, three experienced secondary school writing teachers (≥10 years) participated as expert raters. After a brief calibration session using anchor scripts and the analytic rubric, they independently scored each student’s text without time constraints while blinded to the study hypotheses. Their interrater reliability (two-way random-effects, absolute-agreement ICC for a single measure) was acceptable; ICC (2,1) = 0.71 [0.64, 0.85]. Based on the experts’ grand-mean total score, three level bands were defined—low (10 s), middle (20 s), and high (30 s) out of 40. Within each band, the representative text was identified as the one closest to the band median and whose analytic subscale profile most closely matched the targeted level. The three experts then convened a brief consensus meeting to assign a single agreed set of analytic scores for the selected text at each level. These consensus scores served as the expert reference for computing student teachers’ absolute scoring error. The three representative texts were presented without level labels and in counterbalanced orders across participants. Personally identifying information in the texts was masked prior to scoring and use.

Using the selected texts, student teachers conducted the writing assessment activities as follows. They scored each text with an analytic rubric (Van Den Bergh & Rijlaarsdam, 2001; Kim, 2020) consisting of four 10-point criteria (Appendix C): focus (goal orientation, unity, and clarity of stance), organization (overall structure and cohesion across paragraphs), audience awareness (attention to reader interests and alternative positions), and language use (lexical choice, sentence construction, and grammatical accuracy/appropriateness). Participants then produced feedback based on established guidelines (Parr & Timperley, 2010): they provided one brief overall comment and two specific feedback points for each text (examples in Appendix D). This structured format was prioritized over a more open-ended approach to ensure standardized measurement and comparability across participants, particularly given that student teachers often lack extensive prior experience in generating professional feedback. As every participant evaluated all three texts on four criteria and their feedback was subsequently coded on five dimensions (detailed in Section 2.3.2), this study employed a repeated-measures design (scoring accuracy: 3 × 4, 1932 samples; feedback quality: 3 × 5, 2415 samples).

### 2.3. Measures

#### 2.3.1. Scoring Accuracy

Each participant evaluated three Grade 9 argumentative student essays, representing low-, middle-, and high-proficiency levels, with an average length of 226.33 words per composition. Following established writing assessment research (Engelhard, 1996; Wang et al., 2016, 2017), scoring accuracy was defined as the absolute discrepancy from expert reference scores. In teacher education contexts, deviation from expert judgments serves as an appropriate reference point for evaluating student teachers who have not yet consolidated professional rater expertise, making this criterion particularly relevant for assessing their emerging assessment literacy.

Following prior work (Engelhard, 1996; Wang et al., 2016, 2017), for each participant × text × criterion, the absolute scoring error was calculated as ∣rater score−expert score∣, where smaller values indicate greater accuracy. The absolute reference scores for scoring accuracy were determined by consensus among three expert writing teachers with over 10 years of experience (see Section 2.2 for expert scoring details). It should be noted, however, that these consensus scores represent a professional benchmark derived from an expert panel (ICC = 0.71), rather than an absolute true score. The consensus procedure was implemented as a deliberate methodological step to mitigate idiosyncratic individual biases and to ensure that the reference points reflected a collective professional judgment. Consequently, the accuracy metrics reported herein reflect the degree of alignment with this expert-established reference point. Scores for each analytic criterion ranged from 0 to 10 (total = 40) and were recorded as integers (see Section 2.2 for analytic criteria details).

#### 2.3.2. Feedback Quality

For each student text, the two specific feedback comments written by student teachers were coded across five analytical dimensions following Parr and Timperley’s (2010) framework and point allocation scheme: *evaluation quality (D1), alignment with rubric (D2), accuracy of feedback (D3), presence of actionable suggestions (D4), and textual scope of the comment (D5)*. The specific coding criteria, point allocations, and score ranges for each dimension were defined as follows:

(a) D1 (evaluation quality; range = 0–2 points): Comments that explicitly described how well the student fulfilled the target function, what was needed to meet the goal, and where the current performance diverged from the goal received 2 points (e.g., “Paragraphing is not appropriate; each paragraph should cohere around a single central idea.”). Vague or confusing evaluative statements received 1 point (e.g., “Your main idea could be clearer.”). Comments lacking evaluative substance or relevance received 0 points.
(b) D2 (alignment with rubric; range = 0–1 point, perfect score = 1): received 1 point if the comment mentioned one or more analytic criteria; 0 points otherwise.
(c) D3 (accuracy of feedback; range = 0–1 point, perfect score = 1): received 1 point if the cited evidence and evaluation were appropriate and accurate; 0 points if inaccurate or unclear.
(d) D4 (actionable suggestion; range = 0–1 point, perfect score = 1): received 1 point if the comment included an explicit strategy for improvement (e.g., “After drafting, read your piece aloud to catch errors.”); 0 points if absent or ambiguous.
(e) D5 (textual scope of the comment; range = 0–1 point, perfect score = 1): received 1 point if the comment addressed higher-order, global textual features (rhetorical features, audience, structure, content development, and stylistic voice); 0 points if it addressed only surface-level mechanical features (grammar, spelling, agreement, and precise wording).

After coder training using a shared codebook and reconciling disagreements to consensus, two paid graduate research assistants independently rated all 966 feedback comments (161 participants × 3 texts × 2 comments) over a two-month period. Their final interrater reliability (a two-way random-effects, absolute-agreement ICC for average measures) was satisfactory: D1 = 0.78 [0.70, 0.84]; D2 = 0.79 [0.72, 0.85], D3 = 0.77 [0.70, 0.83], D4 = 0.79 [0.72, 0.84], D5 = 0.73 [0.64, 0.79]. In accordance with Parr and Timperley’s (2010) procedure, for each student text, the scores of the two feedback comments were averaged within each feedback dimension (D1–D5), and the two coders’ ratings were likewise averaged. For the dichotomous dimensions (D2–D5), this averaging procedure transformed discrete binary ratings into a refined 3-point scale (0, 0.5, and 1.0), capturing finer gradations of feedback quality per text. Rather than creating an aggregated composite total score, each of the five dimensions served as an independent outcome variable in subsequent statistical analyses.

#### 2.3.3. Student Teachers’ Empathy

To examine whether student teachers’ empathy interacted with the main results, the Questionnaire of Cognitive and Affective Empathy (QCAE; Reniers et al., 2011) was administered. Cognitive empathy refers to the intellectual ability to adopt and comprehend another person’s perspective, while affective empathy represents the emotional capacity to vicariously experience or respond to the feelings of others. To ensure linguistic and cultural appropriateness, the version of the instrument, which was formally validated (Kang, 2013), was utilized. The QCAE yields these two factors through five subconstructs: for cognitive empathy, perspective taking (10 items) and online simulation (9 items) were used; for affective empathy, emotion contagion (4 items), proximal responsivity (4 items), and peripheral responsivity (4 items) were used. All items were rated on a 1–4 Likert scale. For each participant, raw scores were calculated as the mean rating across relevant items for cognitive empathy (19 items) and affective empathy (12 items), respectively (ranging from 1.0 to 4.0). Prior to statistical modeling, these raw scores were grand-mean centered. This standard transformation was applied to minimize structural multicollinearity between main effects and interaction terms in moderating models and to allow main effects to be meaningfully interpreted at the sample average of empathy. Item reliability within the current sample was α = 0.901 for cognitive empathy and α = 0.841 for affective empathy, indicating excellent and good internal consistency, respectively.

#### 2.3.4. Student Teachers’ Own Writing Quality

Before the scoring/feedback tasks, participants wrote a short argumentative essay to serve as a benchmark of their own writing proficiency. This writing quality variable was employed as a moderator to examine the effectiveness of student teachers’ writing assessment. The essay was scored using the same four analytic criteria from the main assessment study (focus, organization, audience awareness, and language use). The use of identical criteria was intended to ensure theoretical and empirical alignment between the participants’ own performance and the specific assessment tasks they were assigned, thereby allowing for a criterion-to-criterion analysis of how proficiency in a specific domain relates to scoring accuracy within that same domain. Two trained graduate raters evaluated the quality of participants’ essays, and rater blinding ensured that no identifying participant information was accessible. Interrater reliability (a two-way random-effects, absolute-agreement ICC for average measures) was 0.83 [0.71, 0.90] for focus, 0.87 [0.77, 0.93] for organization, 0.76 [0.60, 0.86] for audience awareness, and 0.81 [0.68, 0.89] for language use. The two ratings were averaged, and the resulting raw scores were mean-centered for analysis.

### 2.4. Analysis

The primary aim of the statistical analysis was threefold: (a) to evaluate how student teachers’ scoring accuracy systematically varied across student text proficiency levels (low, middle, and high) and analytic rubric criteria; (b) to evaluate how their written feedback quality systematically varied across student text proficiency levels and feedback dimensions; and (c) to assess whether these task-level relationships were moderated by student teachers’ individual traits (their own writing quality, cognitive empathy, and affective empathy).

To account for the hierarchical and nested structure of the data—specifically, multiple scoring judgments (1932 observations) and feedback segments (2415 coded units) nested within 161 student teachers across three student text proficiency levels—linear mixed-effects models (LMMs) were employed. LMMs are particularly well-suited for repeated-measures assessment data because traditional linear regressions or ANOVAs assume independence of observations, an assumption violated when individual raters evaluate multiple texts across several criteria. Prior to analyses, categorical predictors were coded with sum-to-zero contrasts. Continuous moderators—student teachers’ own writing quality, cognitive empathy, and affective empathy—were standardized (*z*-scores; grand-mean centered and scaled to 1 *SD*) to facilitate interpretation of slopes and interactions. Comparisons of model fit using AIC/BIC and likelihood-ratio tests supported the use of intercept-only structures as reported here, given negligible improvements in fit and occasional singular fits; thus, the more parsimonious models were retained.

For scoring accuracy, the model predicted absolute scoring error from text proficiency level, criterion, student teachers’ writing quality, cognitive empathy, and affective empathy, including their interactions, with random intercepts for rater (ID), ID × criterion, and ID × level (total observations *N* = 1932; 161 participants). For feedback quality, a parallel model predicted the coded quality scores from level, dimension (D1–D5), and the same covariates, with random intercepts for ID, ID × dimension, and ID × level (*N* = 2415; 161 participants). Although dimensions D2 through D5 were originally coded as binary at the individual comment level (0 or 1), averaging ratings across participants’ two comments per text and independent coders produced text-level scores with multiple intermediate values (0, 0.25, 0.5, 0.75, and 1.0). This aggregation expanded outcome variability and approximated continuous distributional properties, justifying their analysis within linear mixed-effects models. However, to ensure robustness against bounded outcome distributions, sensitivity analyses on the feedback quality model were additionally conducted using distribution-specific models: a cumulative link mixed model (CLMM) for D1 and binomial generalized linear mixed models (GLMMs) for D2 through D5.

Omnibus fixed effects were evaluated using Type III sums of squares with Kenward–Roger degrees of freedom. Where appropriate, follow-up comparisons employed Bonferroni-adjusted EMMs for levels within each criterion or dimension. For moderation analysis, simple slopes were examined with emtrends, and slopes across levels were compared. Assumption checks—including normality, homoscedasticity, influential observations, and multicollinearity—were conducted; diagnostics revealed no serious violations and acceptable collinearity.

All analyses were conducted in R 4.5.1 (R Core Team, 2025) using *lme4* for model estimation (Bates et al., 2015), *lmerTest* for Satterthwaite/Kenward–Roger degrees of freedom (Kuznetsova et al., 2017), *emmeans* for estimated marginal means (Lenth, 2024), *effectsize* for partial η^2^ (Ben-Shachar et al., 2020), and *performance* for diagnostics (Lüdecke et al., 2021).

## 3. Results

### 3.1. Preliminary Analysis: Descriptive Statistics and Performance Across Text Proficiency Levels

Prior to conducting multi-variable linear mixed-effects modeling, preliminary descriptive analyses were performed to examine student teachers’ baseline characteristics and their evaluative performance across student text proficiency levels. Student teachers’ individual traits (rater-level covariates) are summarized in Table 1a, including personal writing quality, cognitive empathy, and affective empathy. Descriptive statistics for the primary outcome variables—absolute scoring error and feedback quality dimensions across the low-, middle-, and high-proficiency student texts—are presented in Table 1b.

To address the directional orientation of scoring errors (systematic over- or under-scoring), descriptive signed error metrics (rater score–expert score) were additionally examined across student text proficiency levels and rubric criteria (Table 1c). Single-sample *t*-tests against zero revealed that participants exhibited a pervasive over-scoring (leniency) bias for mid-level texts (*M*_signed_ = +1.39, *SD* = 1.65, *p* < 0.001) and low-level texts (*M*_signed_ = +0.89, *SD* = 2.31, *p* < 0.001). Conversely, high-level texts elicited a general under-scoring (severity) bias (*M*_signed_ = −0.69, *SD* = 1.87, *p* < 0.001).

Criterion-specific analyses further illustrated distinct directional patterns. For low-level texts, over-scoring was overwhelmingly concentrated in the focus criterion (*M*_signed_ = +3.12, *SD* = 2.47), whereas language use showed slight under-scoring (*M*_signed_ = −0.39, *SD* = 1.44). For mid-level texts, over-scoring was present across all four criteria, peaking in focus (*M*_signed_ = +1.80, *SD* = 1.23) and language use (*M*_signed_ = +1.75, *SD* = 1.69). For high-level texts, under-scoring was most severe in audience awareness (*M*_signed_ = −1.63, *SD* = 1.94) and focus (*M*_signed_ = −0.98, *SD* = 1.73), while organization showed no significant directional bias (*M*_signed_ = +0.17, *p* = 0.195).

### 3.2. Differences in Student Teachers’ Scoring Accuracy

As indicated in Table 2, a linear mixed-effects model (LMM) was used to estimate absolute scoring error from student text proficiency level (high, middle, and low), scoring criterion (audience awareness, focus, organization, and language use), and (centered) teacher covariates (student teachers’ writing quality, cognitive empathy, and affective empathy), with random intercepts for rater (ID), ID × criterion, and ID × level (*N* = 1932 observations; 161 participants; 483 ID × level and 644 ID × criterion combinations). In presenting the LMM results below, fixed-effect degrees of freedom (*df*), *F*- and *p*-values, and their effect sizes (partial η^2^) are reported for each specification. Satterthwaite/Kenward–Roger approximations were employed. Although a within-subjects design can theoretically accommodate random slopes, they were excluded from the final model because models incorporating random slopes resulted in over-parameterization. Model selection was therefore guided by likelihood-ratio tests and information criteria (AIC/BIC), prioritizing a more parsimonious random-intercept-only structure to ensure the stability of the fixed-effect estimates. The model sufficiently satisfied assumptions for inference: diagnostic panels showed acceptable linearity, homoscedasticity, and approximate normality of residuals; influential points remained within reference contours. Collinearity was low, with all adjusted VIFs ≤ 1.71 (the highest for interaction terms with writing quality).

Bonferroni-adjusted pairwise comparisons within each scoring criterion revealed clear text proficiency level differences in focus and language use, with more subtle patterns observed in organization and audience awareness. Type III tests (Satterthwaite) found a main effect of level, *F*(2, 382.78) = 6.68, *p* = 0.001; a pronounced effect of criterion, *F*(3, 507.27) = 55.86, *p* < 0.001; and a significant level × criterion interaction, *F*(6, 1014.23) = 44.06, *p* < 0.001.

Effect-size estimates (partial η^2^) were 0.03 (level), 0.25 (criterion), and 0.21 (level × criterion). The main effect of student teachers’ own writing quality was not significant, but there was a small level × writing quality interaction, *F*(2, 1275.00) = 3.28, *p* = 0.038 (partial η^2^ = 0.01). Additionally, cognitive empathy moderated level differences across scoring criteria (three-way level × criterion × cognitive empathy), *F*(6, 964.75) = 2.68, *p* = 0.014 (partial η^2^ = 0.02). No other effects reached significance (all partial η^2^ ≤ 0.01).

**Figure 1:**
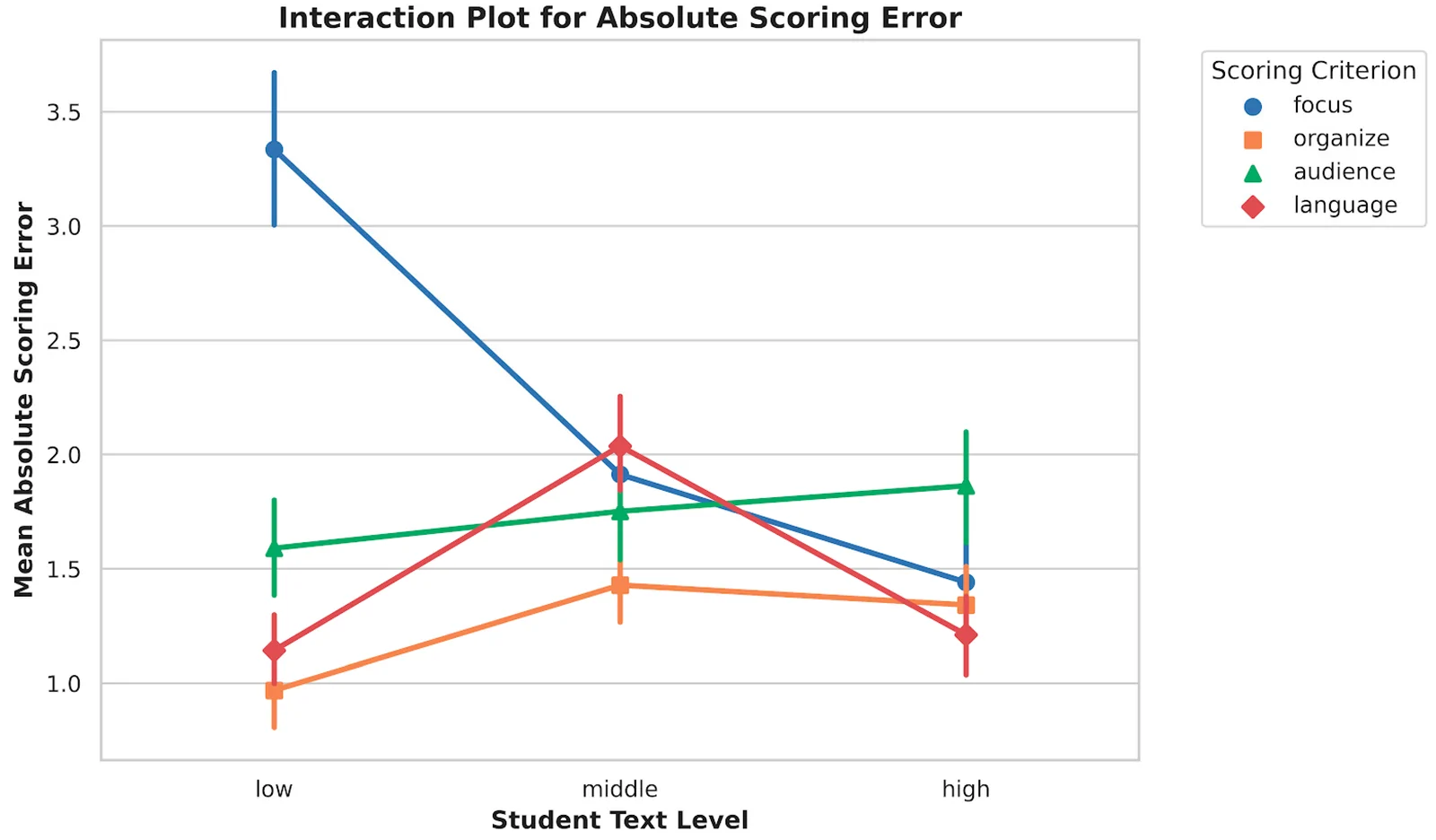
Interaction between student text proficiency level and scoring criterion for absolute scoring error.

Figure 1 illustrates this interaction, revealing how scoring accuracy fluctuates across different criteria depending on student performance levels. The most pronounced scoring errors occurred in the focus criterion for low-level texts.

Follow-up estimated marginal means with Bonferroni adjustments further clarified these criterion-specific patterns across proficiency levels (Table 3):

- Focus: low-level texts resulted in substantially larger scoring errors than either high-level (estimate = −1.95, *p* < 0.001) or middle-level texts (estimate = 1.44, *p* < 0.001);
- Language use: middle-level texts produced significantly greater scoring errors than both high-level (estimate = −0.91, *p* < 0.001) and low-level texts (estimate = −0.89, *p* < 0.001);
- Organization: low-level texts produced slightly smaller scoring errors compared to high-level (estimate = 0.36, *p* = 0.042) and middle-level texts (estimate = −0.47, *p* = 0.004);
- Audience awareness: high-level texts exhibited slightly larger scoring errors than low-level texts (estimate = 0.41, *p* = 0.046).

### 3.3. Differences in Student Teachers’ Feedback Quality

As indicated in Table 4, a second mixed model predicted feedback quality from text proficiency level, feedback dimension (D1–D5), and the same (centered) teacher covariates (writing quality, cognitive empathy, and affective empathy), with random intercepts for ID, ID × dimension, and ID × level (*N* = 2415; 161 raters; 805 ID × dimension; 483 ID × level). Model assumption checks also showed acceptable linearity, homoscedasticity, and approximately normal distribution of residuals; leverage points were within reference contours. Collinearity was low; all adjusted VIFs were ≤ 2.10 (largest for interaction terms with empathy).

Type III tests revealed significant effects of text proficiency level, feedback dimension, and their interaction, as well as effects involving cognitive empathy (main effect and interactions with text proficiency level and feedback dimension). The analyses indicated a small effect of text proficiency level, *F*(2, 322) = 4.23, *p* = 0.015, a very large effect of the feedback dimension, *F*(4, 644) = 736.35, *p* < 0.001, and a robust level × dimension interaction, *F*(8, 1288) = 20.54, *p* < 0.001. Cognitive empathy exhibited a positive main effect, *F*(1, 161) = 4.45, *p* = 0.036, and interacted with text proficiency level, *F*(2, 322) = 3.16, *p* = 0.044, and the feedback dimension, *F*(4, 644) = 2.94, *p* = 0.020. The effect size estimates (partial η^2^) were: level = 0.03, dimension = 0.82, level × dimension = 0.11, cognitive empathy = 0.03, dimension × cognitive empathy = 0.02, and level × cognitive empathy = 0.02. Notably, student teachers’ writing quality was not a significant predictor of their feedback quality (*F* = 1.39, *p* > 0.05).

**Figure 2:**
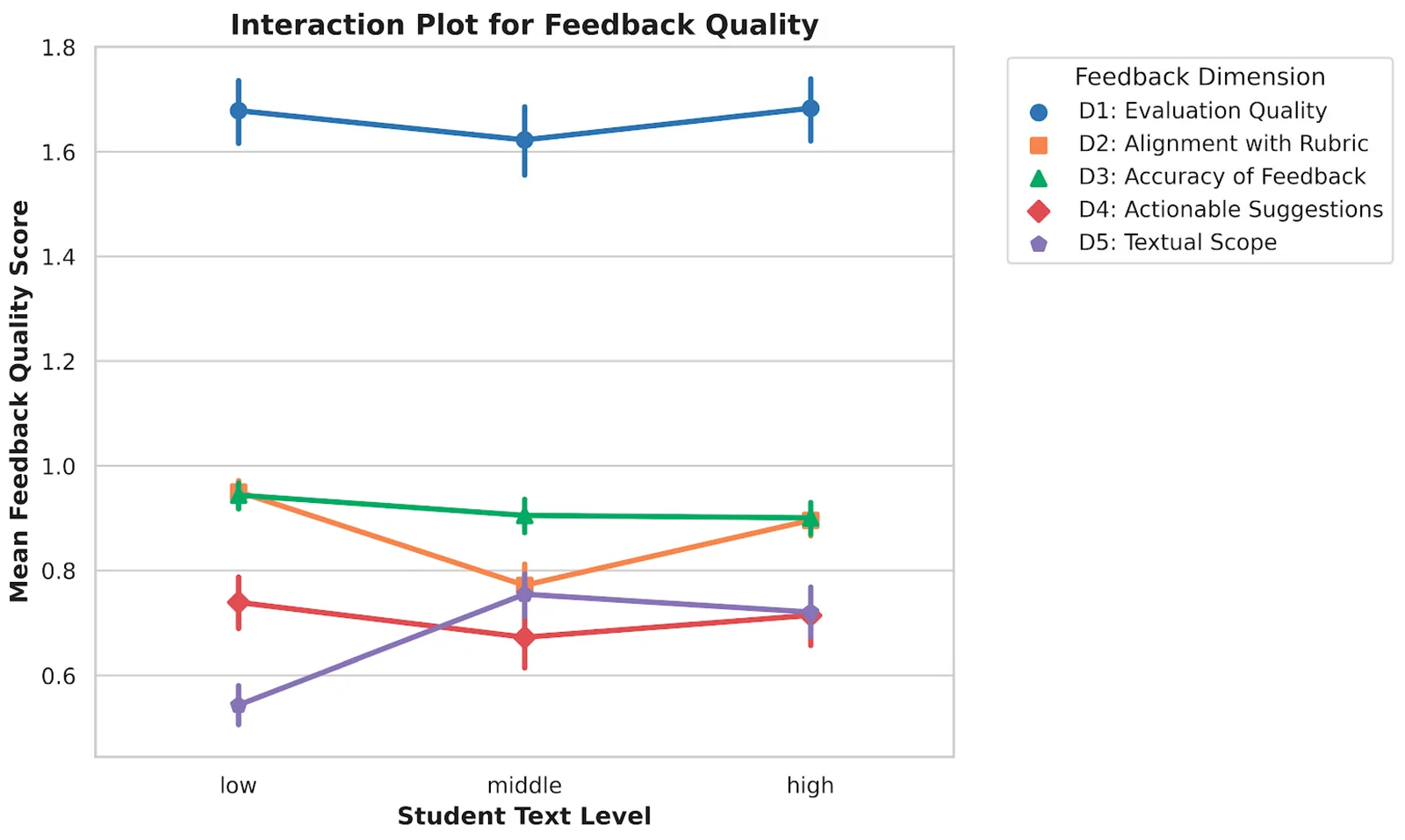
Interaction between student text proficiency level and feedback dimension for feedback quality.

The patterns of feedback quality across these five dimensions are visualized in Figure 2, illustrating a surface-reactive, error-driven tendency in student teachers’ feedback practices. Rather than applying a consistent instructional strategy across all student writers, student teachers’ feedback focus tended to be passively dictated by the visible error density of the evaluated text: while they provided significantly more global and holistic feedback (addressing overall structure, argument development, and rhetorical voice) for high- and middle-level texts, their feedback for low-level texts was predominantly localized (addressing surface grammar, mechanics, and isolated word choice).

Bonferroni-adjusted comparisons within each feedback dimension (Table 5) revealed level differences primarily on D2 and D5. On D1 (evaluation quality), high exceeded middle (*p* = 0.046). On D2 (alignment with rubric), both high and low exceeded middle (both *p*s < 0.001). D3 (accuracy of feedback) did not show significant level differences. On D4 (actionable suggestions), low exceeded middle (*p* = 0.023). On D5 (textual scope of the comment), high and middle exceeded low (both *p*s < 0.001).

Finally, sensitivity analyses employing distribution-specific models (a CLMM for D1 and binomial GLMMs for D2–D5) were evaluated to test the robustness of these primary LMM findings. For D1, D2, D3, and D5, the sensitivity models yielded empirical patterns virtually identical to the primary Gaussian LMMs, confirming that text-level fluctuations were robustly concentrated in rubric alignment (D2; *χ*^2^ = 40.09, *p* < 0.001) and textual scope (D5; *χ*^2^ = 37.71, *p* < 0.001), alongside a modest mid-level drop in evaluation quality (D1; *β* = –0.48, *p* = 0.049). For D4, however, while the Gaussian LMM suggested a weak pairwise contrast (*p* = 0.023), the binomial GLMM omnibus test indicated that the main effect of text level was not statistically robust (*χ*^2^ = 4.50, *p* = 0.105). Taken together, these sensitivity checks confirm that primary LMM inferences regarding feedback quality are robust to outcome scale properties and model specification, while clarifying that D4 exhibits no robust systematic variation across text levels.

### 3.4. Moderation by Student Teachers’ Own Writing Quality and Empathy

Student teachers’ own writing quality did not robustly moderate either outcome. Regarding scoring accuracy, the small level × writing quality term was significant in the omnibus test (Table 2), *F*(2, 1275.00) = 3.28, *p* = 0.038, partial η^2^ = 0.01. However, within-criterion slope comparisons were not significant after correction for multiple comparisons; importantly, text proficiency level gaps persisted across the writing quality continuum (Table 6). For feedback quality, neither level × writing quality nor dimension × writing quality reached significance, and the patterns of text proficiency level differences remained consistent across writing quality (Table 7).

Regarding empathy, affective empathy did not demonstrate consistent associations with either outcome. Its main effects and all interactions with text proficiency level, scoring criterion, and feedback dimension were nonsignificant, and the corresponding partial η^2^ values were close to zero. In contrast, cognitive empathy revealed notable moderation effects. For scoring accuracy (Table 8), the level × criterion × cognitive empathy interaction was significant, *F*(6, 964.75) = 2.68, *p* = 0.014, partial η^2^ = 0.02. Follow-up emtrends attributed this effect to the focus criterion: as cognitive empathy increased, absolute error decreased for high-level texts but increased for low-level texts, resulting in significantly different slopes (high vs. low; high vs. middle). There was no evidence of reliable cognitive empathy moderation for organization, audience, or language use criteria. However, given the small effect size and the nonsignificance of within-criterion slope tests after correction, this interactional effect should be interpreted with caution. It suggests a preliminary, albeit modest, link where higher cognitive empathy might facilitate a more nuanced interpretation of high-level texts while potentially complicating the evaluation of lower-level ones.

For feedback quality (Table 9), cognitive empathy exhibited a significant positive main effect, *F*(1, 161) = 4.45, *p* = 0.036, partial η^2^ = 0.03, and also interacted with text proficiency level and feedback dimension. The interaction was most evident on D1 (evaluation quality), where slope differences were observed across text proficiency levels (high vs. low; low vs. middle), suggesting that empathy-related gains were larger for stronger texts. For other feedback dimensions (D2, D3, D4, and D5), text proficiency level gaps were substantial and consistent, but slope differences by cognitive empathy were not significant.

## 4. Discussion

This study illustrates that student teachers’ assessment performance is dynamically shaped by the interplay between contextual task demands and rater-level individual traits. Synthesizing the empirical findings across the three research questions yields three primary insights. First, evaluative breakdown is most acute not in extreme cases, but in boundary-case, mid-level student sample texts, where ambiguous cues elevate intrinsic difficulties and compromise both scoring accuracy and diagnostic feedback quality. Second, evaluative difficulty shifts dynamically across contexts, as assessment accuracy varies systematically depending on the specific pairing of text level and rubric criteria (e.g., structural criteria in low-level texts versus rhetorical criteria in high-level texts). Third, individual writing proficiency is insufficient to counteract potential evaluative strain; instead, cognitive empathy—specifically perspective-taking—may serve as a protective trait, enabling student teachers to navigate text ambiguity more effectively.

### 4.1. Systematic Differences in Student Teachers’ Scoring Accuracy (RQ1)

Overall, student teachers’ scoring accuracy (i.e., alignment with expert benchmarks) was a behavioral manifestation of rater cognition, heavily dictated by the interaction between task-level criteria and student text proficiency. The significant main effect of rubric criterion and the level × criterion interaction plausibly indicates that evaluating writing imposes criterion-specific difficulties that dynamically shift depending on text quality.

Rather than a uniform difficulty pattern, expert-referenced scoring accuracy revealed distinct evaluative constraints across criteria. For *focus* and *audience awareness*, accuracy was highest in high-level texts, indicating that explicit rhetorical signposting in strong writing allows raters’ mental models to align smoothly with rubric descriptors. Conversely, *organization* yielded the highest accuracy in low-level texts. This asymmetry suggests a dual processing mechanism: student teachers readily detect salient, structural flaws in weak texts via simplified evaluative strategies, but experience difficulties when evaluating subtle, higher-order structural nuances in proficient writing.

A particularly critical vulnerability emerged in mid-level texts—especially regarding *language use*—which generated the lowest expert-referenced scoring accuracy. These findings align with Wolfe et al.’s (2016) difficulty-to-score framework, offering a plausible theoretical interpretation of how the contradictory mix of strengths and weaknesses in the examined mid-range text may heighten rating difficulty. In line with Lumley’s (2002) conceptualization of rating cognition, mid-proficiency prose may heighten the inherent tension between a rater’s intuitive impression and the rigid boundaries of a rubric. Placed near category thresholds, the examined average essay theoretically invites a complex cognitive reconciliation that could complicate raters’ evaluative decision-making.

This evaluative indeterminacy was likely exacerbated by the raters’ novice status. Lacking the consolidated PCK schemata of experienced assessors, student teachers may struggle to resolve text-level ambiguity, often defaulting to narrow linguistic features or lower-level mechanics (i.e., surface conventions such as spelling, punctuation, capitalization, and formatting) at the expense of higher-order rhetorical goals (Barkaoui, 2010, 2011; Thai & Sheehan, 2022). These systematic patterns highlight that scoring training must go beyond general rubric familiarity to explicitly address criterion-specific constraints and boundary-case text evaluations.

Additionally, the descriptive signed error patterns further enrich the theoretical understanding of rater accuracy by distinguishing between leniency and severity biases. The pervasive over-scoring observed in mid-level and low-level texts indicates that student teachers predominantly operate with a leniency bias when evaluating struggling or average writers. Conversely, the mild under-scoring on high-level texts reflects a severity bias or a compression effect at the upper scale boundary—a phenomenon frequently observed among novice raters who hesitate to award top benchmark ratings.

### 4.2. Systematic Differences in Student Teachers’ Feedback Quality (RQ2)

The results on student teachers’ feedback quality indicate that the quality of their comments varies across different aspects of feedback depending on student text proficiency. Rather than providing uniformly thorough guidance across essay levels, student teachers exhibited distinct dimension-specific sensitivities that align with the varying demands of the target text.

A prominent pattern emerged regarding the textual scope (D5) of feedback. Low-level texts elicited a disproportionate concentration of micro-level comments (sub-sentence mechanical corrections) at the expense of macro-level rhetorical development. Faced with severely flawed writing, student teachers frequently adopted a reactive error correction approach. This shift can be interpreted as a potential cognitive simplification strategy: to cope with the heavy processing strain of deciphering poorly written prose, novice raters may default to automated, surface-level corrections rather than engaging in the effortful processing required for global rhetorical growth. As Parr and Timperley (2010) emphasize, effective formative feedback should provide feed-forward guidance that illuminates pathways for future writing development. By prioritizing immediate remedial corrections over macro-level structural goals, student teachers inadvertently reinforce a deficit-based approach for struggling writers.

Conversely, mid-proficiency texts heightened evaluative strain for rubric alignment (D2) and evaluation depth (D1). While clear-cut texts (high or low) allowed raters to easily map text features to rubric criteria, mid-range essays—where strengths and weaknesses are inextricably intertwined—impaired raters’ ability to generate construct-relevant explanations and identify clear performance gaps. Because boundary-case writing presents ambiguous cues, student teachers struggled to ground their evaluations in formal rubric criteria (D2) or articulate diagnostic gaps (D1). This underscores that boundary-case texts hinder not only scoring accuracy (RQ1) but also the diagnostic alignment required for high-quality feedback.

In contrast, actionable suggestions (D4) and feedback accuracy (D3) exhibited no robust systematic variation across text levels once distribution-specific sensitivity models (binomial GLMMs) were applied. Student teachers maintained baseline diagnostic accuracy (D3) and provided concrete next steps (D4) relatively consistently regardless of student text proficiency. Rather than inducing a global breakdown across all feedback dimensions, evaluative strain under text ambiguity is specifically localized in aligning feedback with formal rubric descriptors (D2), expanding textual scope to macro-level rhetorical concerns (D5), and providing diagnostic evaluation depth (D1).

### 4.3. Moderating Effects of Individual Traits (RQ3)

Beyond task-level interactions, the moderation analyses clarify how student teachers’ individual traits cushion or exacerbate the difficulties imposed by text proficiency level ambiguity. Rather than uniformly altering assessment performance, individual characteristics exerted selective moderating effects, offering key theoretical insights into the psychological mechanisms governing writing evaluation.

Surprisingly, student teachers’ own writing quality did not systematically moderate either scoring accuracy or written feedback quality across text proficiency levels. Stronger writers did not achieve significantly higher scoring accuracy, nor did their writing proficiency alter the text-level gaps observed in feedback depth. This empirical decoupling supports the theoretical distinction between content knowledge and pedagogical content knowledge (PCK) in assessment (Carter et al., 2022). Being a proficient writer reflects individual domain performance, whereas evaluating student prose requires specialized assessment PCK—namely, internalizing rubric standards, detecting subtle text-level boundaries, and formulating diagnostic guidance. Consequently, personal writing proficiency alone does not seem to automatically mitigate evaluative difficulty or impart the evaluative schemata needed to navigate ambiguous writing.

In contrast, cognitive empathy—specifically the capacity for perspective-taking (Reniers et al., 2011)—appeared to act as a constructive moderator when raters evaluated the more ambiguous mid-level text. Higher cognitive empathy seemed to cushion the negative impacts of text ambiguity, particularly by supporting written feedback quality in evaluation depth (D1) and facilitating accuracy in identifying structural focus. Assessing boundary-case or average-quality essays can place greater evaluative demands on raters due to intertwined strengths and weaknesses. In these ambiguous contexts, cognitive empathy may function as a helpful interpretive asset: it potentially enables raters to mentally reconstruct a student’s underlying communicative intent, bridging the gap between raw text features and criterion-referenced judgments. Rather than definitively resolving long-standing debate in the literature (e.g., McNamara, 2000; Waung & Highhouse, 1997), these findings offer preliminary empirical insights suggesting that, within this L1 writing assessment context, cognitive empathy does not necessarily introduce subjective leniency bias but may instead support the diagnostic richness of formative feedback—a pattern that should be interpreted cautiously given the modest effect sizes observed.

However, affective empathy—the capacity for automatic emotional resonance—exhibited no significant main or moderating effects on scoring accuracy or feedback quality. This functional dissociation points toward potentially distinct processing requirements in decontextualized writing evaluation. While cognitive empathy involves deliberate, inferential processing to comprehend student intent, affective empathy relies on vicarious emotional responsiveness. In objective, text-based rating tasks lacking direct interpersonal interaction, emotional resonance may provide limited utility for analytical processing or criterion-referenced analysis. Taken together, these findings suggest that assessment literacy requires more than subject matter mastery; teacher education programs may benefit from explicitly fostering cognitive perspective-taking strategies to help novice raters navigate text-level ambiguities and deliver actionable feedback.

### 4.4. Limitations and Future Directions

This study has several limitations that suggest avenues for future research. First, the absence of an expert benchmark limits our ability to distinguish between novice-specific constraints and universal rater constraints. For instance, variability in feedback quality by student level or feedback dimension may be partly—or even largely—shared with practicing teachers. However, this study neither aimed to compare preservice and in-service teachers nor to make claims about their relative standing. From a practical standpoint, any areas of relative weakness—whether absolute or comparative—identify targets for specific training and support. The degree to which professional experience serves as a protective factor against these assessment pitfalls remains an empirical question for future longitudinal or comparative studies.

Second, one of our moderators—student teachers’ own writing quality—was assessed from a single writing sample, which may not fully capture their writing ability. Although this variable served only as an auxiliary variable in the present analyses, future research should more directly investigate the relationship between student teachers’ writing proficiency and their capacity to teach writing.

Third, another methodological limitation of this study lies in the operationalization of student text proficiency levels using a single representative text per level (low, middle, and high). Although a rigorous, expert-led selection procedure was employed to ensure each essay represented the median performance of its proficiency band, relying on one text per level means that the observed level effects cannot be completely disentangled from text-specific characteristics. Consequently, the generalizability of these findings across different writing topics, prompts, and genres should be interpreted with appropriate caution. Future research should expand this design by incorporating multiple student texts per proficiency level to control for text-specific variance and confirm whether the observed rating patterns persist across broader assessment contexts.

Fourth, while directional signed errors were reported descriptively to clarify whether scoring discrepancies stemmed from over- or under-scoring, the linear mixed-effects models did not directly employ signed error as an inferential outcome variable. Future research should build upon these initial findings by incorporating signed error into dynamic, longitudinal inferential designs to model how rater calibration shifts from systematic leniency/severity toward expert consensus over time.

Fifth, methodological limitations regarding the measurement of written feedback quality should also be acknowledged. While adapting Parr and Timperley’s (2010) framework provided an objective and reliable scheme for coding large-scale data, categorizing qualitative comments into discrete analytical dimensions inherently simplifies the qualitative richness and contextual nuances of authentic teacher feedback. Furthermore, while the provided prompt requirement was necessary to maintain experimental standardization across a large sample, it may have restricted the spontaneous, open-ended feedback practices that occur in authentic classroom settings. Future research should incorporate multi-tiered qualitative coding schemes, evaluate holistic feedback literacy constructs, and utilize flexible prompt conditions to better capture the authentic complexity of teacher feedback.

Lastly, a key limitation of this study lies in the trade-off between internal and ecological validity regarding the assessment task environment. To rigorously isolate the interaction mechanisms between task complexity and rater traits, the evaluation protocol was highly structured, providing a standardized 97 min instructional orientation and limiting the evaluation load to three benchmark texts. While this design minimized confounding variance from cumulative fatigue or temporal constraints, it does not fully replicate authentic classroom conditions, where teachers routinely assess large volumes of student writing under time pressure and shifting contextual demands. Task simplification in this study may have mitigated potential evaluative strain, suggesting that the observed scoring inaccuracies and feedback deficiencies represent a conservative baseline of student teachers’ evaluative challenges. In authentic, high-volume grading contexts, cumulative decision fatigue could further exacerbate rater bias and cognitive avoidance. Future research should extend this experimental framework to longitudinal or ecologically authentic classroom settings to examine how extended evaluation tasks and real-time environmental pressures influence evaluative decision-making and feedback quality.

## 5. Conclusions

This study provides behavioral evidence that student teachers’ assessment performance is highly sensitive to the specific features of the examined student texts and evaluative dimensions. Synthesizing the empirical findings across the three sample essays yields three key insights: first, evaluative performance—encompassing both scoring accuracy and feedback quality—exhibited non-linear patterns across text levels, with the mid-level sample essay presenting unique evaluative challenges, plausibly attributable to a lack of prototypical features that may heighten perceived boundary ambiguity. Second, feedback on the low-level sample text displayed a distinct tendency toward localized error correction, a pattern consistent with theoretical accounts of simplified responding under cognitively demanding assessment conditions. Third, cognitive empathy emerged as a potential psychological resource associated with higher pedagogical feedback quality when evaluating ambiguous text features.

Ultimately, teacher education should move beyond general scoring instruction toward evaluative coaching that assists novice raters in navigating the tension and indeterminacy inherent in professional judgment. Grounded in the empirical patterns observed across the three examined texts, this study offers tentative recommendations for teacher educators and curriculum designers seeking to enhance student teachers’ assessment literacy:

- *Boundary-Case Calibration Workshops*: Traditional rater training predominantly relies on clear-cut anchor papers representing polar proficiency levels. However, the current findings regarding performance drops on the mid-level sample essay suggest that training focus needs to extend toward boundary-case calibration. Teacher education programs could reorient calibration sessions around boundary-case essays, offering explicit evaluative modeling on how to reconcile conflicting textual cues and apply rubric thresholds consistently.
- *Scaffolded Diagnostic Evaluation Protocols*: Attempting to process all analytic criteria simultaneously on highly flawed compositions may elevate processing strain, potentially leading raters to revert to localized surface corrections. Thus, training interventions could guide novice raters to adopt a scaffolded evaluation sequence—prioritizing structural features (e.g., organization) on lower-proficiency texts before shifting focus toward higher-order rhetorical dimensions as text quality improves.
- *Socio-Cognitive Perspective-Taking Workshops*: As cognitive empathy—the capacity to infer a writer’s intended meaning—was positively linked to feedback quality, teacher education curricula could incorporate socio-cognitive workshops. Training student teachers to actively adopt the student writer’s perspective may assist them in diagnosing the underlying developmental logic behind student errors, helping transform written feedback from reactive proofreading into constructive, growth-oriented formative dialogue.

## Figures and Tables

**Table 1:** (a) Descriptive statistics for student teachers’ individual traits. (b) Descriptive statistics for evaluative outcomes across student text proficiency levels. (c) One-sample *t*-tests for signed scoring error.

**(a)**						
**Variable**			***M***	***SD***	**Skewness**	**Kurtosis**
Individual characteristics	Cognitive empathy		59.38	7.56	−0.31	0.91
	Affective empathy		35.27	5.89	−0.17	−0.36
	Writing quality (total)		23.61	3.96	−0.56	−0.07
**(b)**						
**Measure/Outcome**			**Low Level *M* (*SD*)**	**Mid Level *M* (*SD*)**	**High Level *M* (*SD*)**	**Overall *M* (*SD*)**
Absolute scoring error (by Criterion)	Focus		3.34 (2.17)	1.91 (1.05)	1.44 (1.37)	2.23 (1.79)
	Organization		0.97 (1.08)	1.43 (1.05)	1.34 (1.04)	1.25 (1.08)
	Audience awareness		1.59 (1.36)	1.75 (1.29)	1.86 (1.72)	1.73 (1.47)
	Language use		1.14 (0.95)	2.04 (1.33)	1.21 (1.10)	1.46 (1.21)
Feedback quality (by Dimension)	Evaluation quality (D1)		1.68 (0.39)	1.62 (0.42)	1.68 (0.38)	1.66 (0.40)
	Alignment with rubric (D2)		0.95 (0.15)	0.77 (0.26)	0.90 (0.19)	0.87 (0.22)
	Accuracy of feedback (D3)		0.94 (0.16)	0.91 (0.21)	0.90 (0.20)	0.92 (0.19)
	Actionable suggestions (D4)		0.74 (0.32)	0.67 (0.37)	0.71 (0.34)	0.71 (0.35)
	Text level of comments (D5)		0.54 (0.24)	0.75 (0.27)	0.72 (0.30)	0.67 (0.29)
**(c)**						
**Text Level**	**Criterion**	**Mean Signed Error**	***SD***	***t***	***p***	**Directional Bias**
Low	Overall	0.89	2.31	9.77	<0.001	Over-scoring
	Focus	3.12	2.47	16.03	<0.001	Over-scoring
	Organization	0.35	1.41	3.12	0.002	Over-scoring
	Audience awareness	0.47	2.04	2.94	0.004	Over-scoring
	Language use	−0.39	1.44	−3.38	<0.001	Under-scoring
Middle	Overall	1.39	1.65	21.4	<0.001	Over-scoring
	Focus	1.80	1.23	18.57	<0.001	Over-scoring
	Organization	1.12	1.38	10.31	<0.001	Over-scoring
	Audience awareness	0.88	1.99	5.62	<0.001	Over-scoring
	Language use	1.75	1.69	13.14	<0.001	Over-scoring
High	Overall	−0.69	1.87	−9.32	<0.001	Under-scoring
	Focus	−0.98	1.73	−7.21	<0.001	Under-scoring
	Organization	0.17	1.69	1.30	0.195	No Bias
	Audience awareness	−1.63	1.94	−10.66	<0.001	Under-scoring
	Language use	−0.32	1.61	−2.50	0.14	Under-scoring

*Note*. (a) Gender counts: 109 women and 52 men. Writing quality refers to the scores for essays written by participants. (b) *N* = 161 student teachers (*n* = 1932 scoring judgments; *n* = 2415 feedback segments). Criterion denotes the specific criterion used for analytic scoring; dimension corresponds to a particular aspect of the feedback quality provided by participants. Absolute scoring error is operationalized as |rater score|—|expert score|. Feedback dimension scores (D1–D5) represent the averaged ratings across the two feedback comments generated per student text. Lower absolute error indicates greater scoring accuracy; higher feedback scores indicate better feedback.

**Table 2:** Mixed-effects omnibus tests for scoring accuracy (significant terms only).

	Fixed Effect				Random Effect		
Parameters	*df*	*F*	*p*	Partial η^2^	Groups	Variance	*SD*
					ID	0.01	0.11
Level	2, 382.78	6.68	0.001	0.03	ID: Level	0.33	0.57
Criterion	3, 507.27	55.86	<0.001	0.25	ID: Criterion	0.03	0.18
Level × Criterion	6, 1014.23	44.06	<0.001	0.21			
Level × Writing quality	2, 1275.00	3.28	0.038	0.01			
Level × Criterion × Cognitive empathy	6, 964.75	2.68	0.014	0.02			

*Note*. The Satterthwaite method was applied to determine degrees of freedom; the numerator and denominator degrees of freedom are listed in sequence; effect sizes are indicated by partial η^2^. Level indicates the writing proficiency level of students whose work was graded (low, middle, and high); criterion refers to the analytical scoring subcriteria (focus, organization, audience awareness, and language use). Model formula: absolute scoring error ~ level × criterion × (writing quality + cognitive empathy + affective empathy) + (1|ID) + (1|ID:criterion) + (1|ID:level).

**Table 3:** Significant Bonferroni-adjusted EMM contrasts for absolute scoring error.

Criterion	Contrast	Estimate [95% CI]	*SE*	*Df*	*t*	*p*
Focus	high–low	−1.95 [−2.37, −1.52]	0.15	1202	−12.94	<0.001
Focus	high–middle	−0.51 [−0.93, −0.09]	0.15	1202	−3.39	0.002
Focus	low–middle	1.44 [1.01, 1.86]	0.15	1202	9.55	<0.001
Organization	high–low	0.36 [0.05, 0.78]	0.15	1189	2.46	0.042
Organization	low–middle	−0.47 [−0.88, −0.06]	0.15	1189	−3.19	0.004
Audience	high–low	0.41 [0.06, 0.88]	0.17	1266	2.42	0.046
Language use	high–middle	−0.91 [−1.39, −0.43]	0.17	1272	−5.32	<0.001
Language use	low–middle	−0.89 [−1.37, −0.41]	0.17	1272	−5.24	<0.001

*Note*. Lower scoring error values correspond to higher accuracy in scoring.

**Table 4:** Mixed-effects omnibus tests for feedback quality (significant terms only).

	Fixed Effect				Random Effect		
Parameters	*df*	*F*	*p*	Partial η^2^	Groups	Variance	*SD*
					ID	0.01	0.11
Level	2, 322	4.23	0.015	0.03	ID: Level	0.01	0.07
Dimension	4, 644	736.35	<0.001	0.82	ID: Dimension	0.02	0.14
Cognitive empathy	1, 161	4.45	0.036	0.03			
Level × Dimension	8, 1288	20.54	<0.001	0.11			
Level × Cognitive empathy	2, 322	3.16	0.044	0.02			
Dimension × Cognitive empathy	4, 644	2.94	0.020	0.02			

*Note*. The Satterthwaite method was used to determine degrees of freedom; the numerator and denominator degrees of freedom are listed in sequence; effect sizes are indicated by partial η^2^. Level indicates the writing proficiency level of students whose work was graded (low, middle, and high); dimension denotes feedback quality subdimensions (evaluation quality, alignment with rubric, accuracy of feedback, actionable suggestions, and textual scope of the comment). Model formula: feedback quality ~ level × dimension × (writing quality + cognitive empathy + affective empathy) + (1|ID) + (1|ID:dimension) + (1|ID:level).

**Table 5:** Significant Bonferroni-adjusted EMM contrasts for feedback quality.

Dimension	Contrast	Estimate [95% CI]	*SE*	*df*	*t*	*p*
D1 (Evaluation quality)	high–middle	0.06 [0.01, 0.15]	0.03	1576	2.43	0.046
D2 (Alignment with rubric)	high–middle	0.12 [0.03, 0.21]	0.03	1576	4.98	<0.001
D2 (Alignment with rubric)	low–middle	0.18 [0.09, 0.27]	0.03	1576	7.16	<0.001
D4 (Actionable suggestions)	low–middle	0.07 [0.02, 0.16]	0.03	1576	2.68	0.023
D5 (Text scope of comments)	high–low	0.18 [0.09, 0.27]	0.03	1576	7.09	<0.001
D5 (Text scope of comments)	low–middle	−0.21 [−0.30, −0.12]	0.03	1576	−8.46	<0.001

**Table 6:** Scoring accuracy: significant conditional contrasts between levels at low and high writing quality (Bonferroni-adjusted).

Criterion	Writing Quality	Contrast	Estimate [95% CI]	*SE*	*df*	*t*	*p*
Focus	−1 *SD*	High–Low	−2.21 [−2.81, −1.61]	0.25	1321	−8.79	<0.001
Focus	−1 *SD*	High–Middle	−0.71 [−1.31, −0.10]	0.25	1321	−2.81	0.015
Focus	−1 *SD*	Low–Middle	1.50 [0.90, 2.11]	0.25	1321	5.98	<0.001
Language use	−1 *SD*	High–Middle	−1.12 [−1.94, −0.29]	0.35	1303	−3.24	0.004
Language use	−1 *SD*	Low–Middle	−0.89 [−1.72, −0.07]	0.35	1303	−2.59	0.029
Organization	−1 *SD*	Low–Middle	−0.57 [−1.12, −0.03]	0.23	1320	−2.51	0.037
Audience	+1 *SD*	High–Low	0.60 [0.01, 1.19]	0.25	1320	2.43	0.046
Focus	+1 *SD*	High–Low	−1.68 [−2.16, −1.20]	0.20	1311	−8.34	<0.001
Focus	+1 *SD*	Low–Middle	1.37 [0.88, 1.85]	0.20	1311	6.78	<0.001
Language use	+1 *SD*	High–Middle	−0.70 [−1.18, −0.22]	0.20	1312	−3.47	0.002
Language use	+1 *SD*	Low–Middle	−0.89 [−1.38, −0.41]	0.20	1312	−4.45	<0.001

**Table 7:** Feedback quality: significant conditional contrasts between levels at low and high writing quality (Bonferroni-adjusted).

Dimension	Writing Quality	Contrast	Estimate [95% CI]	*SE*	*df*	*t*	*p*
D2	−1 *SD*	High–Middle	0.13 [0.05, 0.22]	0.04	1576	3.70	<0.001
D2	−1 *SD*	Low–Middle	0.18 [0.10, 0.26]	0.04	1576	5.10	<0.001
D5	−1 *SD*	High–Low	0.15 [0.06, 0.23]	0.04	1576	4.19	<0.001
D5	−1 *SD*	Low–Middle	−0.17 [−0.25, −0.08]	0.04	1576	−4.67	<0.001
D2	+1 *SD*	High–Middle	0.12 [0.03, 0.20]	0.04	1576	3.33	0.003
D2	+1 *SD*	Low–Middle	0.18 [0.09, 0.26]	0.04	1576	5.01	<0.001
D5	+1 *SD*	High–Low	0.21 [0.12, 0.29]	0.04	1576	5.82	<0.001
D5	+1 *SD*	Low–Middle	−0.26 [−0.34, −0.17]	0.04	1576	−7.28	<0.001

*Note*. D2 = alignment with rubric; D5 = textual scope of comments.

**Table 8:** Scoring accuracy: significant differences in slope for cognitive empathy across levels according to criterion (Bonferroni-adjusted).

Criterion	Slope Contrast	Estimate [95% CI]	*SE*	*df*	*t*	*p*
Focus	High–Low	−0.61 [−0.99, −0.22]	0.16	1185	−3.79	<0.001
Focus	High–Middle	−0.46 [−0.85, −0.08]	0.16	1185	−2.87	0.013

**Table 9:** Feedback quality: significant differences in slope for cognitive empathy across levels according to dimension (Bonferroni-adjusted).

Dimension	Slope Contrast	Estimate [95% CI]	*SE*	*df*	*t*	*p*
D1	High–Low	0.09 [0.03, 0.16]	0.03	1576	3.42	0.002
D1	Low–Middle	−0.08 [−0.15, −0.02]	0.03	1576	−2.97	0.009

*Note*. D1 = evaluation quality.

## Data Availability

The anonymized datasets and R analysis script underlying this article are available on the Open Science Framework (OSF): https://osf.io/k475q/overview?view_only=ca7a5888ef1f4ba7bdc191d7bcaa0e3c (accessed on 5 December 2025).

## Appendix A. Prompt for Writing Scoring and Feedback Task

1. Students’ Writing Task Specifications
  - Task Content: Compose an argumentative essay supporting a stance with valid evidence regarding an issue in school life or educational situations.
  - Target Audience: Grade 9 students (Middle School 3rd Grade)
  - Genre: Argumentative writing
  - Task Context: Intended for publication in an open online space for expressing opinions, such as a personal blog or school bulletin board.
  - Time/Location: 45 min/Computer lab (Word processor writing)
  - Note: As these compositions are authentic Grade 9 student writings presented in their original, unedited form, please approach the scoring and feedback tasks with the mindset of being the student’s actual classroom teacher.
2. Scoring and Feedback Instructions
  - Refer to the analytic scoring rubric and record the score for each criterion in the evaluation table located at the top of each text.
  - Upon completing the scoring, provide written feedback in the bottom margin of each text according to the following procedure:
    (1) First, write a brief overall comment and impression.
    (2) Next, write exactly two essential feedback points that would be most beneficial for improving the student’s writing.
  - Note: While formulating your feedback, you may directly annotate the text or utilize symbols within the student’s writing as necessary.

## Appendix B. Representative Grade 9 Student Essay Samples

**Level**	**Content (Translated from Korean)**
High	A while ago, I saw an article about Seungjeongwon Ilgi (Journals of the Royal Secretariat). It recorded the history of the Joseon Dynasty in great detail, and its sheer volume is said to be so vast that translating the whole text is extremely difficult. Until now, when thinking of Joseon historical records, I only knew about Joseon Wangjo Sillok (Annals of the Joseon Dynasty), but reading this article recently made me realize that our country possesses such a valuable cultural heritage. Although I had heard the name Seungjeongwon Ilgi before, it was a cultural heritage item I did not know much about specifically. These books recorded the daily lives of kings and various national events continuously. Although records for some kings are missing due to unfortunate events in history, the volume of the remaining documents alone is said to be unorganized yet. Unlike Joseon Wangjo Sillok, Seungjeongwon Ilgi has remained relatively unknown to the public. I think the reason lies in our country’s approach to promoting cultural heritage. Because the volume is so vast, publishing it as books for easy public access was inherently difficult even with the translated parts, but it is also true that there was too little promotion of our cultural heritage to the general public. Seungjeongwon Ilgi is a collection of books that shows our ancestors’ passion for record-keeping. In my opinion, our country needs to actively promote and inform both our people and foreign countries about these rich inheritances. We should raise public awareness, pride, and interest in our own heritage, and actively promote our superior culture abroad. I hope the current government shows greater interest in preserving and promoting our cultural heritage.
Mid	These days, on the internet I repeatedly keep seeing bad comments, profanity, and other comments that make the other person feel bad. A while ago I saw a person posted a question on the internet, and there I happened to see by chance that there were more than 10 comments attached. Looking at those comments, I saw people leaving strange words, profanity, etc. Seeing that, I came to think about how bad the person who posted the question would feel. Also there was a time like this too. Someone asked a math question so I replied with an answer, and besides me, another person answered too. But at that time I answered with all the formulas and answers for the math question, but the other person answered with only the answer. And then looking at that person’s comment section, I saw the questioner left a comment saying “lol u don’t know this problem right? No formulas.” And then the person who answered saw that comment and wrote “It’s not that I don’t know okay?” and after that the formal polite language changed to informal language and in the end used profanity. I think these kinds of bad comments and profanity on the internet that make others feel bad must disappear. Almost all netizens think nothing of leaving comments carelessly, and act as if they did nothing wrong. In severe cases among our country’s celebrities…
Low	I think 9th graders should also go on a school trip The reason for going on a school trip in the 1st semester is to help us get closer with friends. Even if we are in 9th grade there could be friends we don’t know and there could be friends we are not close with cause that’s why. So 9th graders also gotta go on 3 days 2 nights school trip ---------------------------------- I think we should get rid off the uniform blazer jacket. Because the blazer is too thin and on top of being cold I think the only advantage is that the pockets are deep. Make the blazer warmer or I think we should be allowed to wear something else instead of the blazer. ---------------------------------- I wish warm hot water came out in the school restroom Because my hands are super freaking cold that I hate washing hands Since I’m lunch duty I gotta wash them but every time I wash my hands are too cold ----------------------------------- In summer I think the AC should be blasted high Because sometimes on the news when students dying appear I get scared

## Appendix C. Analytic Scoring Rubric for Argumentative Writing

	**Focus**	**Organization**	**Audience Awareness**	**Language Use**
9–10	The writer’s stance is exceptionally clear. All parts of the essay are tightly and coherently connected around the central argument.	Demonstrates a complete 3-part argumentative structure (Introduction, Body, Conclusion). Paragraphing, content distinction, and logical sequencing are smooth and appropriate.	Content and expressions are tailored to engage audience interest. Content thoughtfully considers diverse reader perspectives and counter-arguments.	Employs precise, highly appropriate, and engaging vocabulary suited to context. Sentences are clear, easy to comprehend, and display excellent subject-verb/clause agreement.
7–8	The writer’s stance is generally clear. Contains a minor amount of unnecessary or slightly off-topic content.	Possesses a complete argumentative structure, though certain structural stages are slightly inadequate. Paragraphing is somewhat imprecise, but the overall progression is acceptable.	Content and expressions are moderately aligned with reader interest. Fairly acceptable in accommodating general reader perspectives.	Vocabulary usage is generally appropriate. Sentence structure is mostly acceptable, despite occasional vague phrasing or awkward constructions.
5–6	The writer’s stance is sometimes unclear or ambiguous. Contains several irrelevances unaligned with the topic.	Argumentative structure is noticeably deficient. Paragraph distinction is poorly executed, causing minor structural confusion.	Limited consideration of the reader’s perspective, including content and expressions unrelated to the reader’s interests/concerns.	Sometimes uses inappropriate or awkward vocabulary. Some sentences are unclear in meaning or lack agreement.
3–4	The writer’s stance is quite unclear or ambiguous. Contains considerable irrelevances unaligned with the topic.	Lacks a complete structure as a persuasive piece. Paragraph divisions and content order are generally inappropriate.	Almost no content or expressions related to the reader’s interests/concerns. Almost no consideration of the reader’s diverse perspectives	Frequently uses inappropriate or awkward vocabulary. Sentences are often overly complex, disorganized, or lack proper agreement, hindering readability.
1–2	The writer’s stance cannot be determined. The essay as a whole completely strays from the assigned topic.	Fails to display an argumentative essay structure. The sequence of ideas is disorderly and chaotic.	Shows complete disregard for reader interest. Entirely ignores the audience’s perspective.	Vocabulary usage and sentence construction skills are severely deficient. Extremely difficult to discern the intended meaning.
10 points per criterion; Maximum total score = 40 points.				

## Appendix D. Representative Examples of Student Teachers’ Written Feedback

Feedback Example 1:

“It was empathic to read because you pointed out various real-life problems that a 3rd-grade middle school student could feel. However, I see a few disappointing points in OO’s writing, so I would like to talk about 2 main things. First, it’s regrettable that the claim and evidence were not clearly connected. For example, OO clearly claimed in the 2nd paragraph to ‘abolish the school jacket’, but looking at the evidence and the last sentence, it says that the jacket is cold so please make it warm. Besides this, the grounds for other claims are not sufficient, so it feels a bit lacking to include the content in the proposal letter. Second, considering the reader, I think it would be good to slightly fix grammatical elements. How about reading and reviewing it yourself after finishing writing? Things you didn’t know when writing at first, such as incorrect spelling, spacing, and sentence agreement, might be visible if you read it again.”

Feedback Example 2:

“The claim of the whole text is ambiguous and it seems like a text that just listed thoughts in your head. Since multiple positions are mixed in one text, it is difficult to grasp the core content. First, it seems that deciding your own position clearly should be the priority. After that, it would be good to organize and restructure the content according to your position to write a unified text.”
